# Association Between Cd Exposure and Risk of Prostate Cancer: A PRISMA-Compliant Systematic Review and Meta-Analysis

**DOI:** 10.1097/MD.0000000000002708

**Published:** 2016-02-12

**Authors:** Song Ju-Kun, Dong-Bo Yuan, Hao-Fu Rao, Tian-Fei Chen, Bo-Shi Luan, Xiao-Ming Xu, Fu-Neng Jiang, Wei-De Zhong, Jian-Guo Zhu

**Affiliations:** From the Department of Oral and maxillofacial surgery (SJ-K), Guizhou Provincial People's Hospital, Guiyang, Guizhou; Department of Urology (D-BY, H-FR, T-FC, B-SL, J-GZ), Guizhou Provincial People's Hospital, Guizhou, Guiyang; Department of Urology (X-MX), Ningbo No. 2 Hospital, Ningbo; Department of Urology (F-NJ, W-DZ, J-GZ), Guangdong Key Laboratory of Clinical Molecular Medicine and Diagnostics, Guangzhou First People's Hospital, Guangzhou Medical University, Guiyang, Guangzhou; and Urology Key Laboratory of Guangdong Province (F-NJ, W-DZ, J-GZ), The First Affiliated, Hospital of Guangzhou Medical University, Guangzhou Medical University, Guangzhou, China.

## Abstract

Several observational studies on the association between Cd exposure and risk of prostate cancer have yielded inconsistent results. To address this issue, we conducted a meta-analysis to evaluate the correlation between Cd exposure and risk of prostate cancer.

Relevant studies in PubMed and Embase databases were retrieved until October 2015. We compared the highest and lowest meta-analyses to quantitatively evaluate the relationship between Cd exposure and risk of prostate cancer. Summary estimates were obtained using a random-effects model.

In the general population, high Cd exposure was not associated with increased prostate cancer (OR 1.21; 95% CI 0.91–1.64), whereas the combined standardized mortality ratio of the association between Cd exposure and risk of prostate cancer was 1.66 (95% CI 1.10–2.50) in populations exposed to occupational Cd. In addition, high D-Cd intake (OR 1.07; 95% CI 0.96–1.20) and U-Cd concentration (OR 0.86; 95% CI 0.48–1.55) among the general population was not related to the increased risk of prostate cancer. In the dose analysis, the summary relative risk was 1.07 (95% CI 0.73–1.57) for each 0.5 μg/g creatinine increase in U-Cd and 1.02 (95% CI 0.99–1.06) for each 10 μg/day increase of dietary Cd intake. However, compared with nonoccupational exposure, high occupational Cd exposure may be associated with the increased risk of prostate cancer.

This meta-analysis suggests high Cd exposure as a risk factor for prostate cancer in occupational rather than nonoccupational populations. However, these results should be carefully interpreted because of the significant heterogeneity among studies. Additional large-scale and high-quality prospective studies are needed to confirm the association between Cd exposure and risk of prostate cancer.

## INTRODUCTION

Prostate cancer is one of the most common malignancies in developed countries and is the second most common cancer in men, following lung cancer, worldwide.^1,2^ The incidence and mortality rates of prostate cancer vary markedly among different ethnic groups, with the lowest rates found in China and other parts of Asia and the highest rates detected in Western populations.^3,4^ These differences are caused by genetic susceptibility, exposure to unknown external risk factors, differences in health care and cancer registration, or a combination of these factors. In 2015, up to 220,800 men were diagnosed with prostate cancer, and 27,540 men will die of it in the United States.^2^ In recent decades, a rapid increase in prostate cancer incidence has been observed in fast-developing countries, in which lifestyles have significantly changed. The etiology of prostate cancer comprises multiple factors. Some causative risk factors for prostate cancer have been implicated, including obesity, androgen, and exposure to selenium, lycopene, vitamins D and E, dietary fat, and Cd.^5–7^

Cd is a minor metal found naturally in the earth's crust and has been widely distributed in the environment as a result of anthropogenic activity. Cd presents an elimination half-life of 10 to 30 years and may exert a wide range of negative effects on human health.^8^ Besides being a carcinogen,^9^ Cd exposure is associated with osteoporosis and bone fracture,^10,11^ type 2 diabetes,^12^ kidney disease,^13^ and cardiovascular disease.^14,15^ Cd is currently one of the most extensive occupational and environmental pollutants. Occupational Cd exposure is used in various industries, such as Cd-emitting industries and metal mines. Occupational Cd exposure occurs when dust and fumes are inhaled. In particular, major sources of natural and anthropogenic Cd in the general population include cigarette smoking and diet choices: tobacco, grains, potatoes, and vegetables taking up Cd from the soil.^16^ Several epidemiologic studies investigating the association between Cd exposure and susceptibility to prostate cancer have yielded inconsistent findings. Some studies have demonstrated a significant correlation^17–22^ or little association^23–25^ between Cd exposure and risk of prostate cancer, but others failed to show any significant association.^26–39^ Therefore, we systematically performed a meta-analysis by combining all available data from observational studies to evaluate the association between Cd exposure and risk of fracture. Our meta-analysis followed the Preferred Reporting Items for Systematic Reviews.^40^

## METHODS

This article presents a systematic review and meta-analysis of previously published studies; therefore, ethical approval and written informed consent from patients are not required. This research was conducted in accordance with the Preferred Reporting Items for Systematic Reviews and Meta-Analysis statement.^40^

### Data Source and Search Strategy

We searched the PubMed and Embase databases until October 2015 to identify relevant studies that evaluated the association between Cd exposure and prostate cancer risk. We used the following search terminologies: “prostate carcinoma” OR “prostatic cancer” OR “prostate cancer” OR “prostatic carcinoma” combined with “Cadmium.” The search was limited to human subjects. Moreover, we manually searched the reference lists of previous reviews and related article references to identify other potentially eligible studies.

### Eligibility Criteria and Study Selection

The inclusion criteria were as follows: (1) Cd was the heavy metal used for exposure to humans; (2) the outcome of interest was prostate cancer incidence, prevalence, and mortality; (3) the report was a cohort, case-control study; and (4) the relative risk (RR), odds ratio (OR), hazard risk, or standardized mortality ratio (SMR) with corresponding 95% confidence interval (CI) was reported or calculated from available data. If the study was reported more than once, we included the study with the most comprehensive data.

### Data Extraction and Quality Assessment

Two authors (JKS and DBY) separately extracted data from selected studies, and discrepancies were resolved through discussion and consensus. For each study, we extracted the first author's name, year of publication, study design, country, total number of cases and subjects, sex, exposure type of Cd, and adjusted variables. When more than 1 adjusted OR was reported, the OR with the most fully adjusted model was selected. For dose analysis, the number of cases and participants of person-years for each category of Cd exposure must also be provided (or data available for calculations).

We evaluated the methodological quality of the included studies by using the Newcastle–Ottawa scale (NOS).^41^ The checklist contained 9 items for case-control studies and cohort studies, with every item accounting for 1 point. We considered high-quality studies as those with a score of >5.

### Statistical Analysis

Differences were expressed as OR with 95% CI for nonoccupational exposure studies and SMR with 95% CI for occupational exposure studies. Prostate cancer caused by Cd was considered a rare event, and the RR in the cohort study was considered as approximations of OR. Three studies reported stratified risk estimates by age^23^ and region.^19,38^ We combined these estimates by using a random-effects model and used the pooled estimates for the meta-analysis. The OR in 1 study^34^ was not extracted; thus, we computed the crude risk estimates and their corresponding CI. A random-effects model of the DerSimonian and Laird method was used to calculate the summary risk estimates, irrespective of heterogeneity, which incorporated both within-study and between-study variability.^42^ Subgroup analysis was stratified by geographic region, study design, quality of NOS scale, type of outcome, and type of exposure. We conducted sensitivity analyses by omitting 1 study in each turn to investigate whether the results were attributed to 1 large study or a study with extreme results. Furthermore, we explored the heterogeneity of the different variables mentioned above through a single-variable meta-regression analysis. We conducted a 2-stage random-effects dose-response meta-analysis by using the method proposed by Greenland and Longnecker.^43^ This method required that the distribution of cases, person-years, non-cases, and risk estimates within the variance are known for at least 3 quantitative exposure categories. We assigned the median values or middle point of Cd exposure for each category to the corresponding RR. If the highest category of the studies was open-ended, we assumed the range to be the same as the adjacent interval. First, we estimated a restricted cubic spline model via generalized least-square regression with 4 knots at 5%, 35%, 65%, and 95% distribution. Second, we pooled the study-specific risk estimates by using the restricted maximum likelihood method in a random-effects meta-analysis. Nonlinear relation was estimated by testing the null hypothesis, which indicated that the coefficient of the second spline is equal to 0.

We also evaluated the potential publication bias by using funnel plot and Egger tests, with a priori *P* < 0.1 indicating a significant publication.^44^ If asymmetry evidence was detected, the trim-and-fill method was employed to correct the publication bias.^45^ All statistical analyses were conducted using Stata version 13.1 (Stata Corp, College Station, TX).

## RESULTS

### Literature Search

**FIGURE 1:**
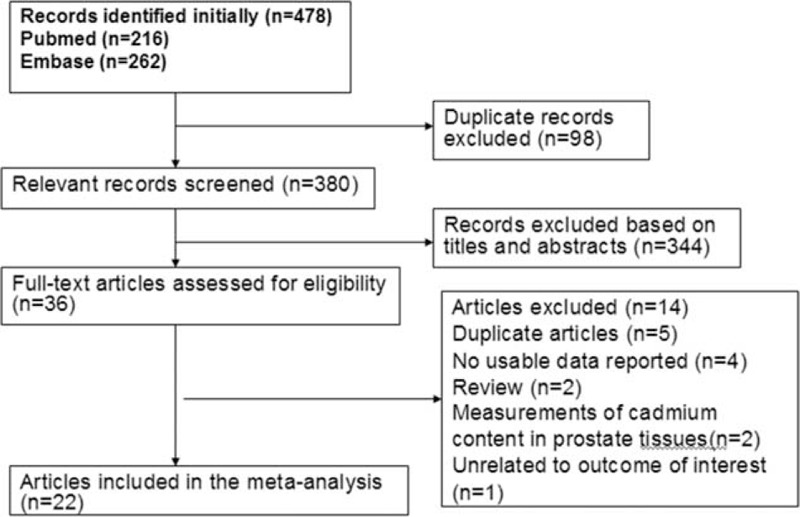
Flow diagram of the literature search and study selection.

Figure 1 shows a flow chart of the inclusion criteria. Following the development of our search strategy, we identified 478 records from PubMed and Embase databases. After excluding the duplicates and articles that did not meet the inclusion criteria, we obtained 36 articles with full-texts read for further evaluation. Five articles were duplicate publications,^17,46–49^ 4 articles presented no data useful for the meta-analysis,^50–53^ 2 articles were excluded because the Cd contents in prostate tissues were measured,^54,55^ 2 articles were reviews,^10,56^ and 1 article reported an association between Cd and prostate-specific antigen.^57^ Finally, 22 studies that met the meta-analysis criteria were included.

### Study Characteristics

Tables 1–3 present the characteristics of the included studies. A total of 22 studies, comprising 8 case-control and 14 cohort studies, contributed to the meta-analysis. These studies were published from 1967 to 2015. The number of prostate cancer patients ranged from 40 to 358 in the case-control studies and from 3 to 83,085 in the cohort studies. Thirteen studies were conducted in Europe,^18–22,29,30,32,35–39^ 7 in the United States,^23,24,26,27,31,33,34^ and 2 in Asia.^25,28^ Thirteen studies reported findings for prostate cancer incidence,^18,20,22–25,28,33–37,39^ whereas the remaining 9 studies reported results for prostate cancer mortality.^19,21,26,27,29–32,38^ We included a total of 200 prostate cancer deaths, 6653 prostate cancer cases, and 137,998 participants in the meta-analysis. Eight studies were designed to evaluate OR,^20,23–25,33–36^ 3 evaluated RR,^18,37,39^ 3 evaluated hazard risk,^26–28^ and 8 evaluated SMR^19,21,22,29–32,38^. Three articles used urinary Cd (U-Cd) as biomarker for long-term exposure to Cd,^25–27^ 4 articles evaluated Cd levels by estimating the dietary Cd (D-Cd) by using food frequency questionnaires,^18,23,28,39^ and 2 studies examined Cd in toenails^20,33^. Twelve studies reported an association between occupational Cd exposure and prostate cancer risk,^19,21,22,24,29–32,34–38^ whereas 10 studies used nonoccupational populations.^18,20,23,25–28,33,36,39^ Most of the studies were controlled for some conventional risk factors, including age (n = 6) and smoking (n = 6). Some studies were also controlled for body mass index (n = 3) and alcohol consumption (n = 2), but few studies were adjusted for beef intake, dairy product consumption (n = 1), and intake of vegetable and fruit (n = 1). None of the studies were adjusted for other heavy metals, trace elements of organic pollutants, and intake of grains.

**TABLE 1:**
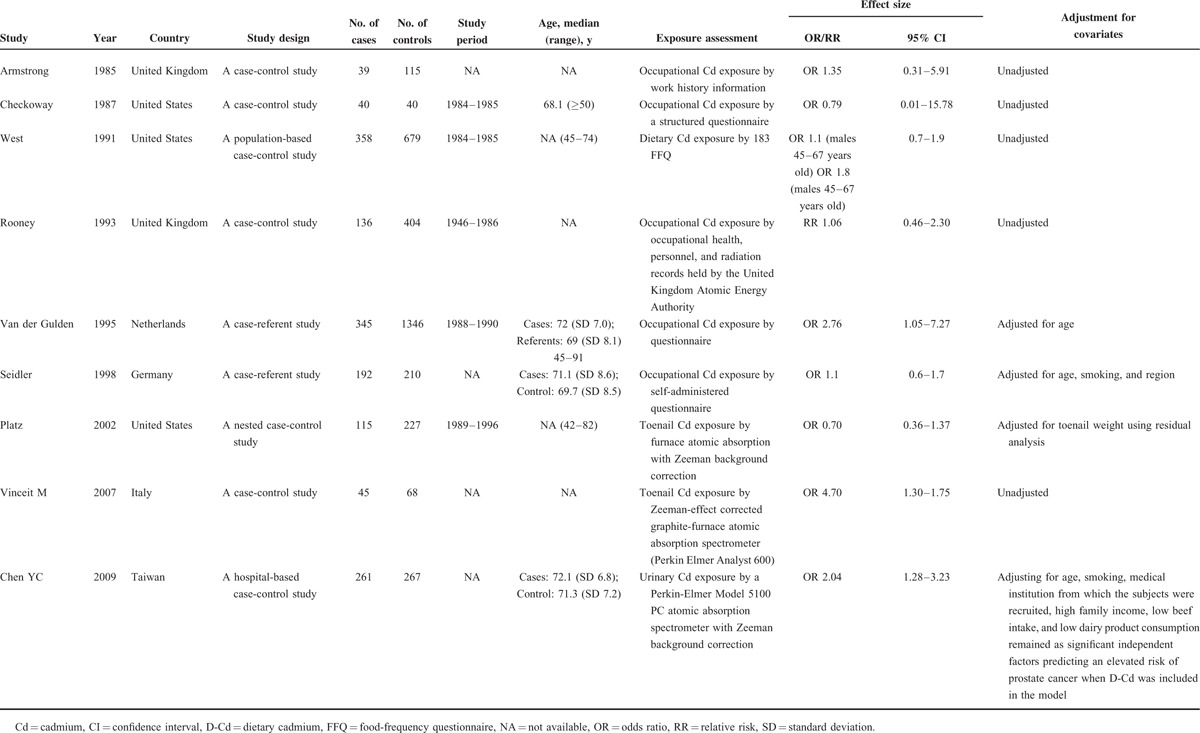
ORs/RR of prostate cancer between patients with and without environmental/occupational Cd exposure among included case-control studies

**TABLE 2:**
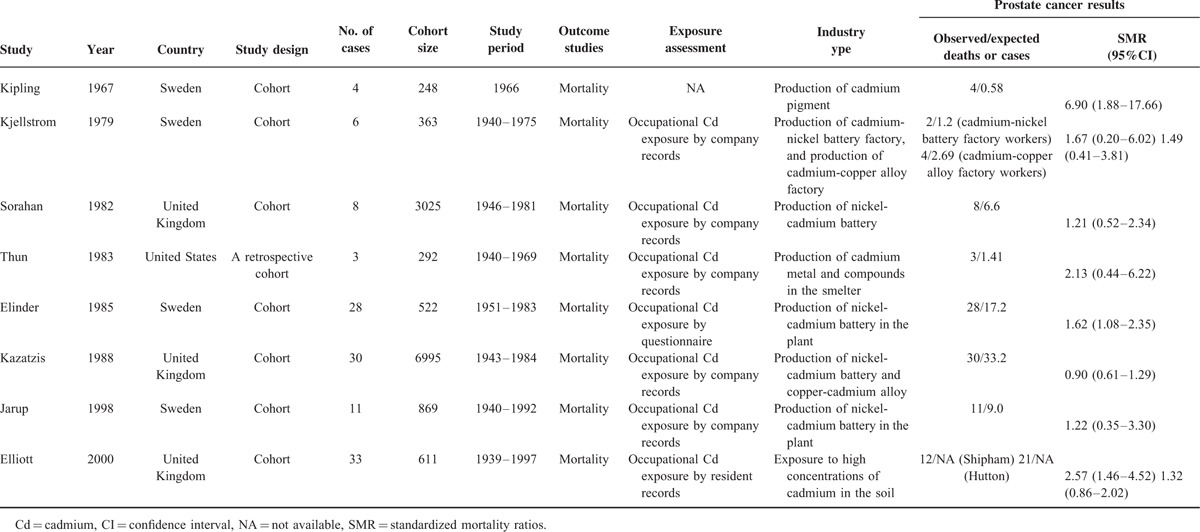
Standardized mortality ratios of prostate cancer patients with and without occupational Cd exposure among included cohort studies

**TABLE 3:**
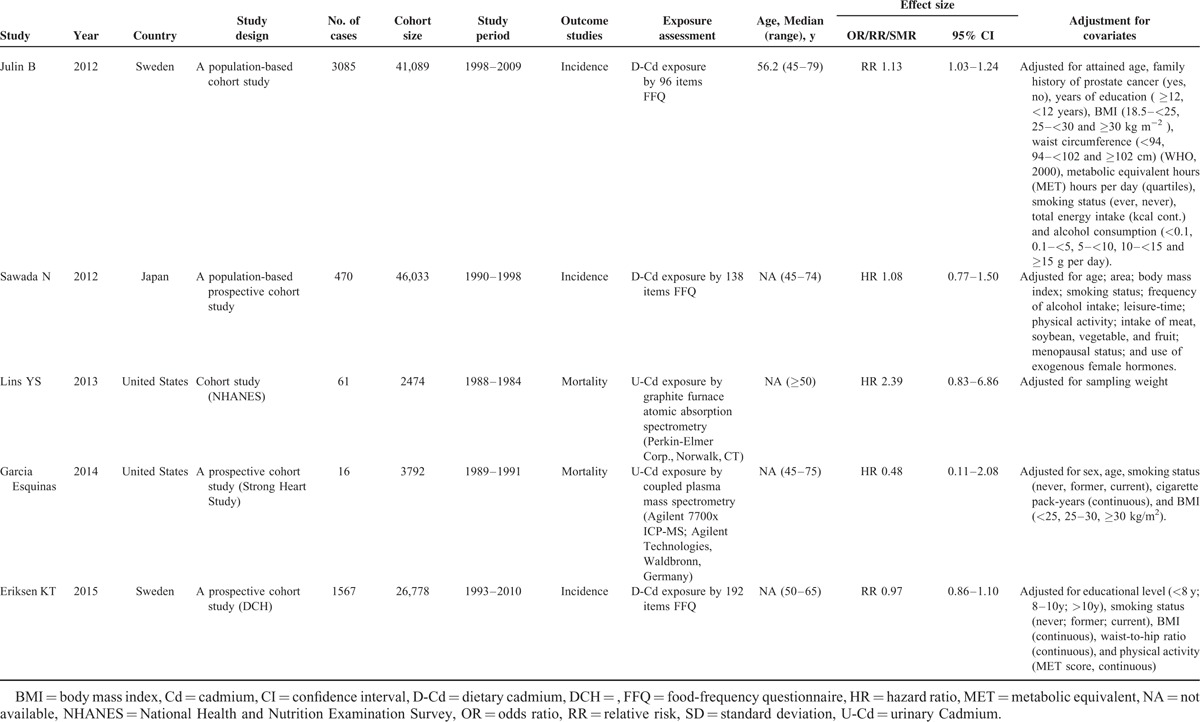
OR/RR/HR of prostate cancer between patients with and without environmental/occupational Cd exposure among included cohort studies

NOS was used to assess the quality of studies included in the meta-analysis (Table 4). The median NOS score was 4.8 (range 3–7).

**TABLE 4:**
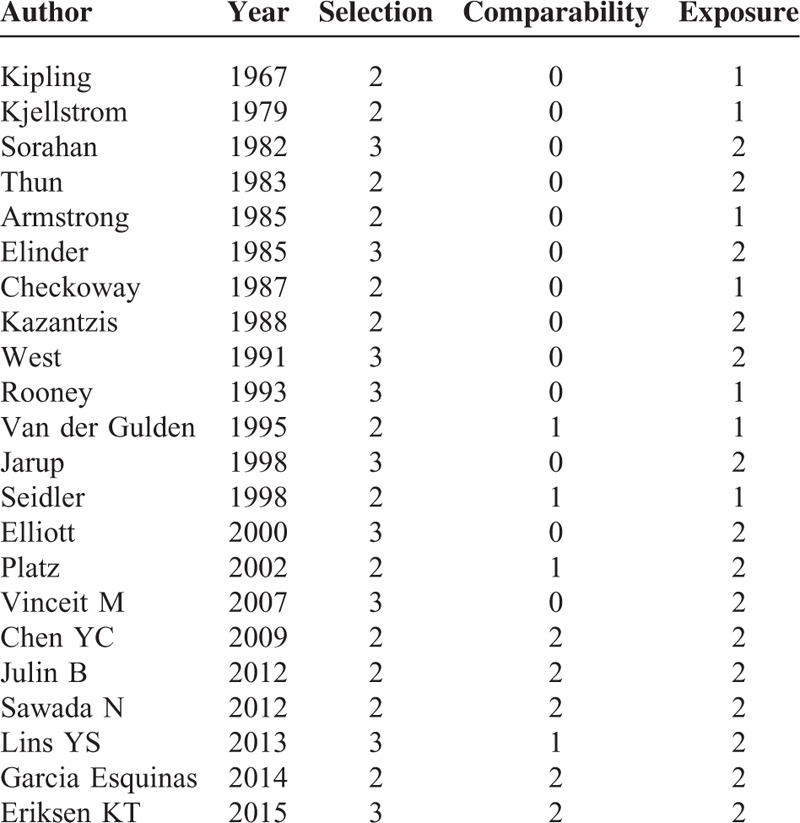
Quality assessment of eligible studies based on Newcastle–Ottawa scale

### Results From Pooled SMR Estimates With and Without Population Exposed to Occupational Cd

**FIGURE 2:**
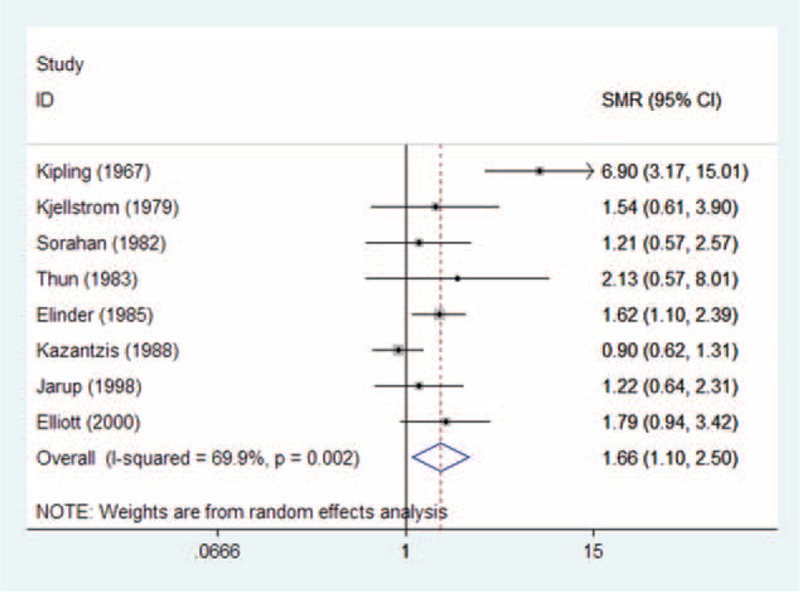
Forest plot of Cd exposure and prostate cancer risk (SMR) in occupational Cd exposure population. Cd = cadmium, SMR = standardized mortality ratio.

**FIGURE 3:**
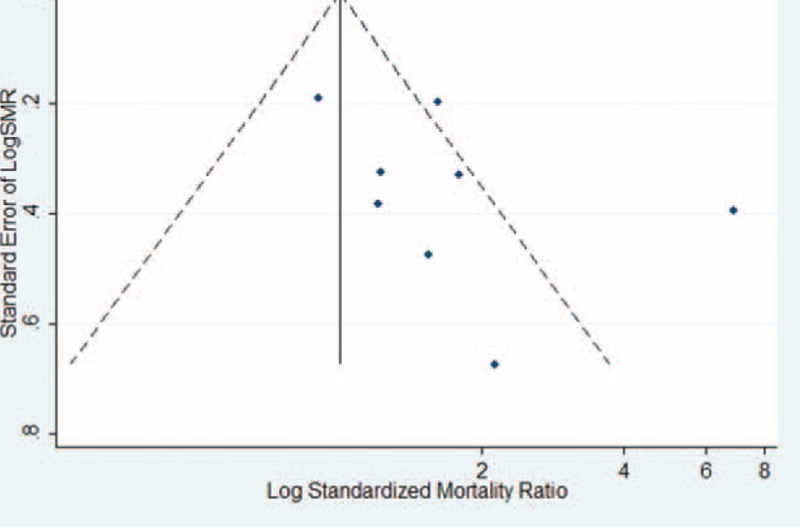
Funnel plot of Cd exposure and prostate cancer risk (SMR) in occupational Cd exposure population. Cd = cadmium, SMR = standardized mortality ratio.

Figure 2 shows the SMR estimates and 95% CI from each study, as well as the pooled SMR estimate based on a random-effects model. Results from the 8 cohort studies indicated that the pooled SMR was 1.66 (95% CI 1.10–2.50) with moderate heterogeneity (*P*_for_._heterogeneity_ = 0.002; *I*^2^ = 69.9%). In subgroup analyses for exposure type, we restricted each analysis to 7 occupational exposure studies, resulting in a summary SMR of prostate cancer of 1.61 (95% CI 1.04–2.48). Only 1 study was conducted in the United States,^31^ and the 7 other studies were conducted in Europe. When we stratified the analysis by geographic region, the pooled SMR was 1.63 (95% CI 1.05–2.52) for studies conducted in Europe. Compared with a low NOS score (SMR = 2.08, 95% CI 0.73–5.91), the association was higher among studies with high NOS score (OR = 1.51, 95% CI 1.14–1.98) (Table 5). In a sensitivity analysis, similar results were observed, which ranged from 1.30 (95% CI 1.03–1.64) with low heterogeneity (*I*^2^ = 9.0%, heterogeneity *P* = 0.360) (excluding the study by Kipling et al^22^) to 1.87 (95% CI 1.23–2.83) with significant heterogeneity (*I*^2^ = 58.4%, heterogeneity *P* = 0.025) (excluding the study by Kazantzis et al^30^). Egger test (*P* = 0.241) and funnel plot (Figure 3) showed no publication bias.

**TABLE 5:**
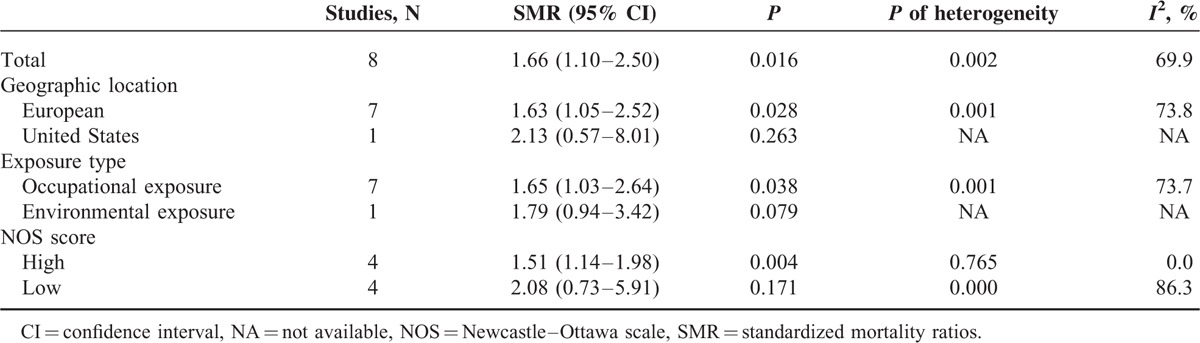
Results of overall subgroup analysis among occupational Cd exposure populations

### Results From Pooled OR Estimates With and Without Environmental/Occupational Cd-Exposed Population

**FIGURE 4:**
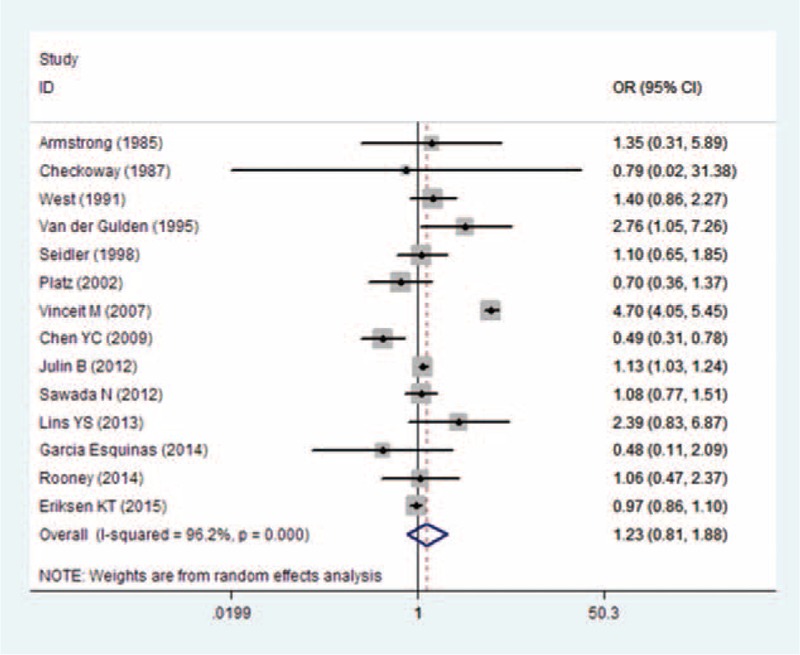
Forest plot of Cd exposure and prostate cancer risk (OR) in occupational/environmental Cd exposure population. Cd = cadmium, OR = odds ratio.

**FIGURE 5:**
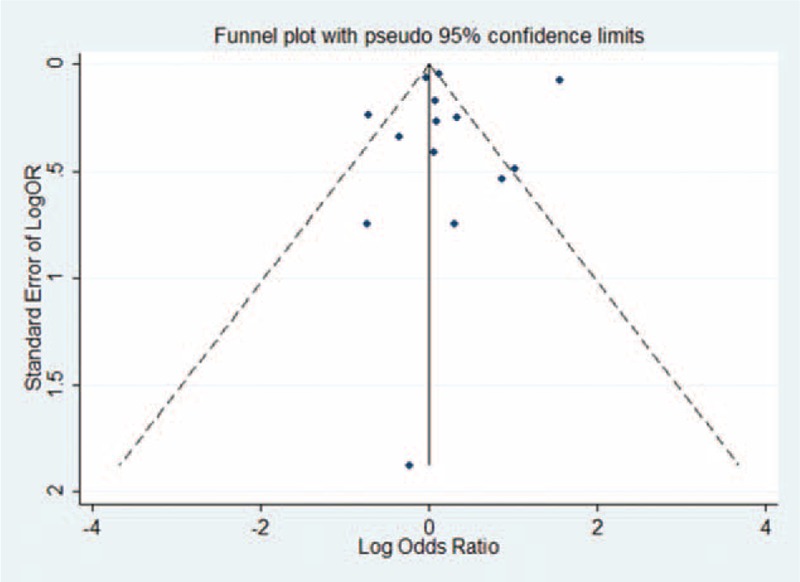
Funnel plot of Cd exposure and prostate cancer risk (OR) in occupational/environmental Cd exposure population. Cd = cadmium, OR = odds ratio.

Figure 4 shows the OR estimates, 95% CI from individual studies, and pooled OR estimate based on a random-effects model. Results from the 14 studies, comprising 9 case-control studies and 6 cohort studies, indicated that the pooled OR was 1.23 (95% CI 0.81–1.88) with significant heterogeneity (*P*_for_._heterogeneity_ = 0.000; *I*^2^ = 96.2%). In subgroup analyses for study design, we restricted each analysis to 9 case-control studies and 5 cohort studies; the summary ORs of prostate cancer for the highest category of Cd exposure versus the lowest category were 1.31 (95% CI 0.60–2.87) and 1.06 (95% CI 0.99–1.09), respectively. Five studies were conducted in the United States, 6 in Europe, and 2 in Asia. When we stratified the analysis by geographic region, the combined OR was 1.10 (95% CI 0.66–1.84) in studies conducted in the United States, 1.57 (95% CI 0.86–2.84) in studies conducted in Europe, and 0.74 (95% CI 0.34–1.60) in studies conducted in Asia. When stratified by type of Cd exposure, the combined OR of prostate cancer was 1.05 (95% CI 0.97–1.15) for D-Cd, 1.18 (95% CI 0.28–2.34) for U-Cd, and 1.87 (95% CI 0.29–12.06) for toenail Cd. Five studies reported an association between occupational Cd exposure and prostate cancer risk; however, the association was not significant in the occupational exposure population (OR = 1.31, 95% CI 0.79–2.19). When stratified by type of outcome, the combined OR was 1.23 (95% CI 0.78–1.95) for prostate cancer incidence and 1.29 (95% CI 0.51–3.27) for prostate cancer mortality. Compared with studies that presented low NOS scores (OR = 1.27, 95% CI 0.87–1.875), the association was higher among studies with high NOS scores (OR = 1.18, 95% CI 0.71–1.96) (Table 6). Six studies^18,25,26,28,35,39^ were adjusted for smoking status, resulting in pooled OR of 0.96 (95% CI 0.80–1.16) with moderate heterogeneity (*P*_for__heterogeneity_ = 0.007; *I*^2^ = 68.8%). Sensitivity analysis showed that the overall pooled estimate was not altered substantially, with the exclusion of 1 study. The overall combined OR after sequential exclusion of 1 study at a time ranged from 1.03 (95% CI 0.88–1.21) with significant heterogeneity (*P*_for heterogeneity_ = 0.017; *I*^2^ = 51.3%) (excluding the study by Vinceit et al^20^) to 1.29 (95% CI, 0.84–1.98) with significant heterogeneity (*P*_for heterogeneity_ = 0.000; *I*^2^ = 96.5%) (excluding the study by Garcia Esquinas et al ^26^). No evidence of publication bias resulted from Egger test (*P* = 0.881) and near-symmetric funnel plot (Figure 5).

**TABLE 6:**
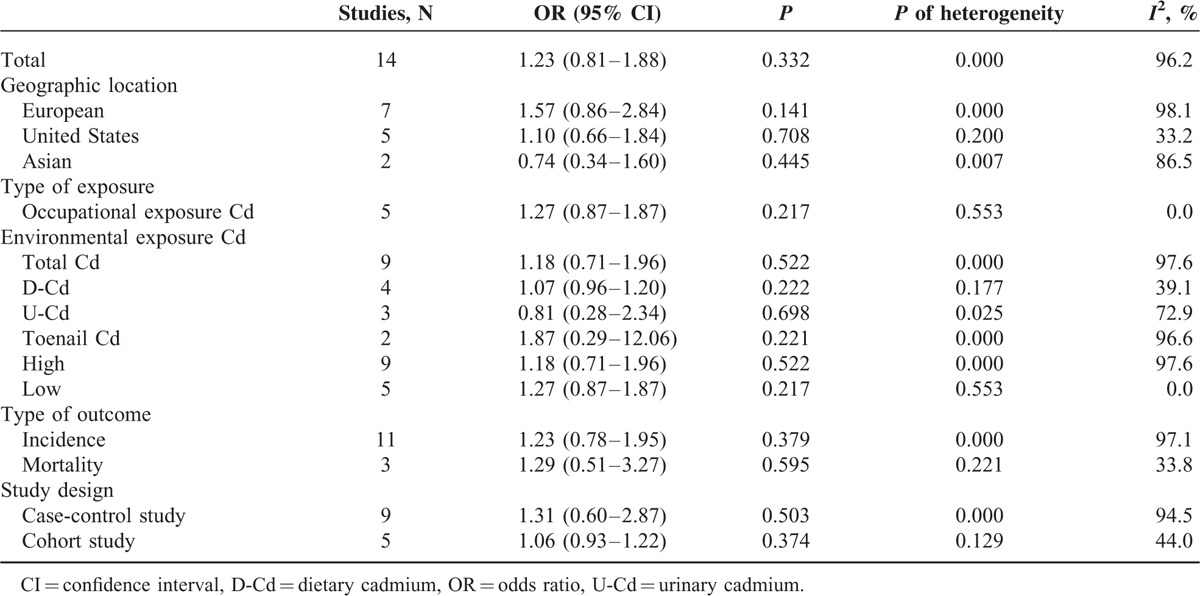
Results of overall subgroup analysis among environmental/occupational Cd exposure populations

Considering the relatively high heterogeneity observed in the trials, a meta-regression was performed to explore the predefined possible sources of heterogeneity. None of the regression coefficients were statistically significant (Table 7), suggesting that publication year, study design, geographic region, NOS, type of outcome, and type of Cd exposure were insignificant sources of heterogeneity.

**TABLE 7:**
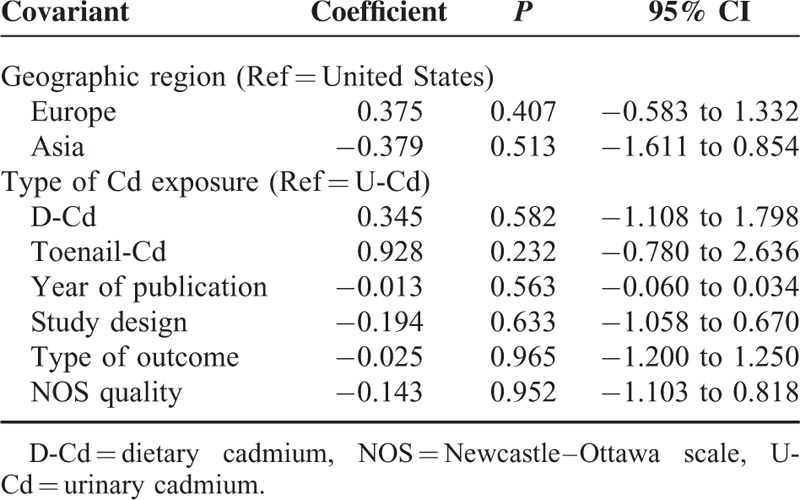
Effects of study variables on meta-regression

**FIGURE 6:**
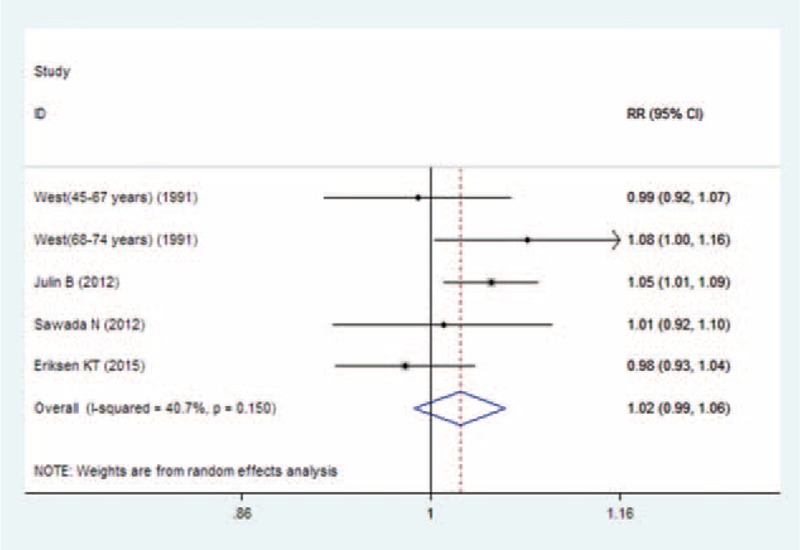
Forest plot of linear trend between dietary Cd intake and risk of prostate cancer (RR), with dose scale of 10 μg/d increase in environmental Cd exposure population. Cd = cadmium, RR = relative risk.

Two studies were included in the dose-response analysis of U-Cd exposure and prostate cancer risk, and a nonlinear association was not observed between them (*P*_for nonlinearitytest_ = 0.47). The summary RR per 0.5 μg/g creatinine increment was 1.07 (95% CI 0.73–1.57) with no evidence of heterogeneity (*P*_for heterogeneity_ = 0.33; *I*^2^ = 0.0%). Four studies were included in the dose-response analysis of D-Cd intake and prostate cancer risk. We found no significant departure from a simple linear-response association between Cd exposure and prostate cancer (*P*_for nonlinearity test_ = 0.64). The estimated RR of prostate cancer risk was 1.02 (95% CI 0.99–1.06) for 10 μg/d increase of D-Cd, with little evidence of heterogeneity (*P*_for heterogeneity_ = 0.15; *I*^2^ = 40.7%) (Figure 6).

## DISCUSSION

Cd is a nonessential metal widely distributed in the environment by industrial and agricultural activities.^8^ According to accumulated evidence from experimental and epidemiologic studies, Cd has been recognized as a human carcinogen.^58,59^ Recently, increasing evidence established a link between Cd exposure and prostate cancer,^33,39^ breast cancer,^60–62^ pancreatic cancer,^63,64^ and lung cancer.^65,66^ Substantial compelling evidence supported that occupational exposure to Cd resulted in lung cancer and showed similar findings regarding prostate and other cancers. A positive association between Cd exposure and prostate cancer mortality was found in 2 cohort studies^21,22^ and 1 case-control study,^36^ whereas several studies showed no significant association among occupational populations.^19,24,29–32,34–38^ These studies were conducted in a Cd-polluted area (eg, nickel batteries, pigments, and soldering alloys). Most nonoccupational Cd exposure studies,^23,26–28,33,39^ though not all,^17,18,20^ showed no significant relation between Cd exposure and prostate cancer risk. Verougstraete et al^67^ conducted a systematic review on Cd-exposed workers and found that Cd exposure was not associated with increased risk of prostate cancer. In another recent meta-analysis based on 8 previous studies, in which D-Cd intake showed no statistically significant association with cancer risk except in stratified analysis by geographic region, a positive association between D-Cd intake and cancer risk was observed.^56^ However, few studies were included in the meta-analysis, which limited the possibility of drawing robust conclusions, especially in the subgroup analysis. Compared with the 2 previous meta-analyses, the current research presented a more extensive systematic review, which included a large number of studies with more than 6828 cases, 123 deaths, and almost 171,972 participants. Thus, we obtained adequate statistical data to clarify the relation between Cd exposure and risk of prostate cancer. In our meta-analysis, we found a positive association between high Cd exposure and risk of prostate cancer for occupational exposure, but not for nonoccupational exposure. These findings can potentially result in higher Cd exposure levels prevailing in these studies.

No substantial changes were observed in most subgroup analyses because the Cd concentrations in the blood and urine are the most common biomarkers for Cd exposure. U-Cd mainly reflects Cd accumulation in the kidney, as determined by lifelong exposure, whereas D-Cd demonstrates a combination of both current and historical exposure. Results from subgroup analyses stratified by type of Cd exposure showed that both U-Cd and D-Cd were not associated with increased prostate cancer risk. Smoking is a primary source of exposure in the general population and is known to damage health through direct and indirect effects. Thus, we also performed subgroup analyses among studies controlled for smoking status to minimize possible non–Cd-mediated negative effects of tobacco smoking on prostate cancer risk. Six publications were adjusted for smoking status, and the results showed that Cd exposure was not associated with increased prostate cancer risk (OR 0.94, 95% CI 0.77–1.14).

Many studies have demonstrated that the prostate is a target organ for the deposition of Cd,^68,69^ and numerous experimental studies in vivo and in vitro have indicated that Cd can act as a prostate carcinogen in rats.^70^ Cd has been recognized as a human carcinogen by the International Agency for Research on Cancer on the basis of mechanistic and epidemiologic evidence from high-exposure occupational settings.^9^ Several mechanisms are responsible for the carcinogenesis of Cd exposure, including induction of oxidative stress,^71^ suppression of DNA repair,^72^ alterations of DNA methylation,^73^ inhibition of apoptosis proto-oncogene activation,^74^ tumor suppressor gene inactivation, and cell adhesion disruption.^75^ In addition, Cd can exert estrogenic activities that play a role in the development of prostate cancer. Cd is suggested to exert estrogenic properties, and direct receptor-mediated effects of estrogen on prostate are plausible.^76^ Experimental evidence showed that excessive exposure to estrogens can cause prostate cancer. In human prostate epithelial cells, Cd exhibits estrogenic activity, including proliferation of prostate cells and activation of the estrogen receptor-α.^77^ Substantial evidence showed a positive relation between Cd exposure and risk of endometrial^78^ and breast cancers.^79^ Therefore, high Cd exposure can potentially increase the risk of prostate cancer.

The present meta-analysis exhibited several strengths. The first research highlight of this meta-analysis is its large sample size. The large number of total cases provided high statistical power to quantitatively evaluate the association between Cd exposure and prostate cancer risk. Second, publication bias is a potential concern in any meta-analysis because small studies with null results do not get published. However, in our meta-analysis, we found little evidence of publication bias.

Nevertheless, some limitations should be considered in the present meta-analysis. First, observational studies, even if prospective, cannot prove causality. We cannot exclude the possibility that the observed positive relationship between Cd exposure and prostate cancer risk is attributed to confounding factors. Majority of the studies were adjusted for potential confounding factors, but not all potential confounders were adjusted in every study. For instance, a potential confounder such as cigarette smoking is not only a source of Cd but also contains other substances with adverse health effects. In analyses stratified by adjusting the smoking status, similar results were obtained. Most studies were adjusted for some conventional risk factors, including age and smoking status, and some studies were controlled for body mass index and alcohol consumption. However, few studies were adjusted for other dietary variables or nutrients, whereas none of the included studies were controlled for other heavy metals, trace elements, or organic pollutants. Second, an accurate assessment of Cd exposure remains a challenge. Most studies used questionnaires to assess Cd exposure, whereas some research used interviews, company records, and self-reports to evaluate Cd concentration. However, increasing errors in measurements become inevitable. The imprecise measurement of Cd concentration might have attenuated the true associations. Third, the definition of Cd exposure varied across studies. The Cd exposure types differed according to geographical locations, as urine Cd concentration (in μg/g creatinine) ranged from approximately 0.39 to 1.46 in the US and European population. In Asian studies, the mean urine Cd concentration (in μg/g creatinine) ranged from 0.94 to 1.4. The Cd intake from food generally varies between 9 and 25 μg/d in the US and in Europe. In Asian studies, the mean D-Cd intake (in μg/day) ranged from 19.7 to 35.4. These factors can affect our results. However, our subgroup analyses showed that the associations between Cd exposure and prostate cancer risk did not differ significantly in terms of study location. Third, potential sources of between-study heterogeneity, which is common in meta-analyses, should be explored. In sensitivity analyses, the observed heterogeneity was explained by an article^22^ that reported a significant positive association and yielded a low NOS. Results from subgroup analyses indicated that geographic region, study design, quality of NOS, type of outcome, and type of exposure are potential sources of heterogeneity. Nevertheless, we used meta-regression and sensitivity analysis to explore the potential causes of between-study heterogeneity. Our meta-regression analysis did not find covariates of publication year, study design, geographic region, NOS, type of outcome, and type of Cd exposure as sources of heterogeneity. Finally, although we selected the highest multivariable-adjusted effect estimates in our meta-analysis, we cannot exclude the possibility that the observed increase in association between Cd exposure and prostate cancer risk among occupational populations can be ascribed to unmeasured or residual confounding factors. The unstable results were observed in occupational and environmental populations, indicating that more relevant articles are needed to further explore this association.

In summary, this meta-analysis suggests high Cd exposure as a potential risk factor for prostate cancer in occupational populations but not in nonoccupational populations. However, these results should be carefully interpreted because of the significant heterogeneity among studies. Additional large-scale and high-quality prospective studies are needed to confirm the association between Cd exposure and risk of prostate cancer.

## References

[1] Jemal A Bray F Center MM Global cancer statistics. *CA Cancer J Clin* 2011; 61:69–90. 21296855 10.3322/caac.20107

[2] Siegel RL Miller KD Jemal A Cancer statistics, 2015. *CA Cancer J Clin* 2015; 65:5–29. 25559415 10.3322/caac.21254

[3] Bashir MN Epidemiology of prostate cancer. *APJCP* 2015; 16:5137–5141. 26225642 10.7314/apjcp.2015.16.13.5137

[4] Zhu Y Wang HK Qu YY Prostate cancer in East Asia: evolving trend over the last decade. *Asian J Androl* 2015; 17:48–57. 25080928 10.4103/1008-682X.132780 PMC4291877

[5] Allott EH Masko EM Freedland SJ Obesity and prostate cancer: weighing the evidence. *Eur Urol* 2013; 63:800–809. 23219374 10.1016/j.eururo.2012.11.013 PMC3597763

[6] Venkateswaran V Klotz LH Diet and prostate cancer: mechanisms of action and implications for chemoprevention. *Nat Rev Urol* 2010; 7:442–453. 20647991 10.1038/nrurol.2010.102

[7] Chan JM Giovannucci EL Vegetables, fruits, associated micronutrients, and risk of prostate cancer. *Epidemiol Rev* 2001; 23:82–86. 11588858 10.1093/oxfordjournals.epirev.a000799

[8] Jarup L Akesson A Current status of Cd as an environmental health problem. *Toxicol Appl Pharmacol* 2009; 238:201–208. 19409405 10.1016/j.taap.2009.04.020

[9] Beryllium, Cadmium, mercury exposures in the glass manufacturing industry. Working Group views and expert opinions, Lyon, 9–16 February 1993. *IARC monographs on the evaluation of carcinogenic risks to humans/World Health Organization. International Agency for Research on Cancer* 1993;58: 1–415. PMC7681476 8022054

[10] Nawrot TS Staessen JA Roels HA Cadmium exposure in the population: from health risks to strategies of prevention. *Biometals* 2010; 23:769–782. 20517707 10.1007/s10534-010-9343-z

[11] Dahl C Sogaard AJ Tell GS Do cadmium, lead, and aluminum in drinking water increase the risk of hip fractures? A NOREPOS study. *Biol Trace Elem Res* 2014; 157:14–23. 24287706 10.1007/s12011-013-9862-x

[12] Barregard L Bergstrom G Fagerberg B Cadmium, type 2 diabetes, and kidney damage in a cohort of middle-aged women. *Environ Res* 2014; 135:311–316. 25462681 10.1016/j.envres.2014.09.017

[13] Byber K Lison D Verougstraete V Cadmium or cadmium compounds and chronic kidney disease in workers and the general population: a systematic review. *Critical reviews in toxicology* 2015; 29:1–50. 10.3109/10408444.2015.1076375 26513605

[14] Julin B Bergkvist C Wolk A Cadmium in diet and risk of cardiovascular disease in women. *Epidemiology* 2013; 24:880–885. 24030503 10.1097/EDE.0b013e3182a777c9

[15] Tellez-Plaza M Jones MR Dominguez-Lucas A Cadmium exposure and clinical cardiovascular disease: a systematic review. *Current atherosclerosis reports* 2013; doi:10.1097/EDE.0b013e31828b0631. 10.1007/s11883-013-0356-2 PMC3858820 23955722

[16] Amzal B Julin B Vahter M Population toxicokinetic modeling of cadmium for health risk assessment. *Environ Health Perspect* 2009; 117:1293–1301. 19672411 10.1289/ehp.0800317 PMC2721875

[17] Cheung MR Kang J Ouyang D Association between urinary Cd and all cause, all cancer and prostate cancer specific mortalities for men: an analysis of national health and nutrition examination survey (NHANES III) data. *Asian Pacific J Cancer Prev* 2014; 15:483–488. 10.7314/apjcp.2014.15.1.483 24528078

[18] Julin B Wolk A Johansson JE Dietary cadmium exposure and prostate cancer incidence: a population-based prospective cohort study. *Br J Cancer* 2012; 107:895–900. 22850555 10.1038/bjc.2012.311 PMC3425979

[19] Kjellstrom T Friberg L Rahnster B Mortality and cancer morbidity among cadmium-exposed workers. *Environ Health Perspect* 1979; 28:199–204. 488034 10.1289/ehp.28-1637490 PMC1637490

[20] Vinceti M Venturelli M Sighinolfi C Case-control study of toenail cadmium and prostate cancer risk in Italy. *Sci Total Environ* 2007; 373:77–81. 17175009 10.1016/j.scitotenv.2006.11.005

[21] Elinder CG Kjellstrom T Hogstedt C Cancer mortality of cadmium workers. *Br J Ind Med* 1985; 42:651–655. 4041382 10.1136/oem.42.10.651 PMC1007553

[22] Kipling M Waterhouse J Cadmium and prostatic carcinoma. *Lancet* 1967; 289:730–731.

[23] West DW Slattery ML Robison LM Adult dietary intake and prostate cancer risk in Utah: a case-control study with special emphasis on aggressive tumors. *Cancer Causes Control* 1991; 2:85–94. 1873441 10.1007/BF00053126

[24] Armstrong BG Kazantzis G Prostatic cancer and chronic respiratory and renal disease in British Cd workers: a case control study. *Br J Ind Med* 1985; 42:540–545. 4016005 10.1136/oem.42.8.540 PMC1007524

[25] Chen YC Pu YS Wu HC Cadmium burden and the risk and phenotype of prostate cancer. *BMC Cancer* 2009; 9:429. 20003241 10.1186/1471-2407-9-429 PMC2797022

[26] Garcia-Esquinas E Pollan M Tellez-Plaza M Cadmium exposure and cancer mortality in a prospective cohort: the strong heart study. *Environ Health Perspect* 2014; 122:363–370. 24531129 10.1289/ehp.1306587 PMC3984227

[27] Lin YS Caffrey JL Lin JW Increased risk of cancer mortality associated with Cd exposures in older Americans with low zinc intake. *J Toxicol Environ Health* 2013; 76:1–15. 10.1080/15287394.2012.722185 23151207

[28] Sawada N Iwasaki M Inoue M Long-term dietary cadmium intake and cancer incidence. *Epidemiology* 2012; 23:368–376. 22415110 10.1097/EDE.0b013e31824d063c

[29] Jarup L Bellander T Hogstedt C Mortality and cancer incidence in Swedish battery workers exposed to cadmium and nickel. *Occup Environ Med* 1998; 55:755–759. 9924452 10.1136/oem.55.11.755 PMC1757526

[30] Kazantzis G Lam TH Sullivan KR Mortality of cadmium-exposed workers. A five-year update. *Scand J Work Environ Health* 1988; 14:220–223. 3175553 10.5271/sjweh.1929

[31] Thun MJ Schnorr TM Smith AB Mortality among a cohort of U.S. Cadmium production workers—an update. *J Natl Cancer Inst* 1985; 74:325–333. 3856046

[32] Sorahan T Waterhouse JA Mortality study of nickel-cadmium battery workers by the method of regression models in life tables. *Br J Ind Med* 1983; 40:293–300. 6871118 10.1136/oem.40.3.293 PMC1069325

[33] Platz EA Helzlsouer KJ Hoffman SC Prediagnostic toenail cadmium and zinc and subsequent prostate cancer risk. *Prostate* 2002; 52:288–296. 12210489 10.1002/pros.10115

[34] Checkoway H DiFerdinando G Hulka BS Medical, life-style, and occupational risk factors for prostate cancer. *Prostate* 1987; 10:79–88. 2434937 10.1002/pros.2990100111

[35] Seidler A Heiskel H Bickeboller R Association between diesel exposure at work and prostate cancer. *Scand J Work Environ Health* 1998; 24:486–494. 9988091 10.5271/sjweh.373

[36] van der Gulden JW Kolk JJ Verbeek AL Work environment and prostate cancer risk. *Prostate* 1995; 27:250–257. 7479392 10.1002/pros.2990270504

[37] Rooney C Beral V Maconochie N Case-control study of prostatic cancer in employees of the United Kingdom Atomic Energy Authority. *BMJ* 1993; 307:1391–1397. 8274891 10.1136/bmj.307.6916.1391 PMC1679658

[38] Elliott P Arnold R Cockings S Risk of mortality, cancer incidence, and stroke in a population potentially exposed to cadmium. *Occup Environ Med* 2000; 57:94–97. 10711276 10.1136/oem.57.2.94 PMC1739911

[39] Eriksen KT Halkjaer J Meliker JR Dietary cadmium intake and risk of prostate cancer: a Danish prospective cohort study. *BMC Cancer* 2015; 15:177. 25884961 10.1186/s12885-015-1153-9 PMC4397739

[R40] Moher D Liberati A Tetzlaff J Group P. Preferred reporting items for systematic reviews and meta-analyses: the PRISMA statement. *BMJ* 2009; 339: b2535. PMC3090117 21603045

[41] Hartling L Milne A Hamm MP Testing the Newcastle Ottawa Scale showed low reliability between individual reviewers. *J Clin Epidemiol* 2013; 66:982–993. 23683848 10.1016/j.jclinepi.2013.03.003

[42] DerSimonian R Laird N Meta-analysis in clinical trials. *Control Clin Trials* 1986; 7:177–188. 3802833 10.1016/0197-2456(86)90046-2

[43] Greenland S Longnecker MP Methods for trend estimation from summarized dose-response data, with applications to meta-analysis. *Am J Epidemiol* 1992; 135:1301–1309. 1626547 10.1093/oxfordjournals.aje.a116237

[44] Egger M Davey Smith G Schneider M Bias in meta-analysis detected by a simple, graphical test. *BMJ* 1997; 315:629–634. 9310563 10.1136/bmj.315.7109.629 PMC2127453

[45] Peters JL Sutton AJ Jones DR Performance of the trim and fill method in the presence of publication bias and between-study heterogeneity. *Stat Med* 2007; 26:4544–4562. 17476644 10.1002/sim.2889

[46] Elghany NA Schumacher MC Slattery ML Occupation, cadmium exposure, and prostate cancer. *Epidemiology* 1990; 1:107–115. 2073496 10.1097/00001648-199003000-00005

[47] Lemen RA Lee JS Wagoner JK Cancer mortality among Cd production workers. *Annals N Y Acad Sci* 1976; 271:273–279. 10.1111/j.1749-6632.1976.tb23122.x 1069514

[48] Armstrong BG Kazantzis G The mortality of Cd workers. *Lancet* 1983; 1:1425–1427. 6134185 10.1016/s0140-6736(83)92368-1

[49] Inskip H Beral V McDowall M Mortality of Shipham residents: 40-year follow-up. *Lancet* 1982; 1:896–899. 6122112 10.1016/s0140-6736(82)92163-8

[50] Ross RK Shimizu H Paganini-Hill A Case-control studies of prostate cancer in blacks and whites in southern California. *J Natl Cancer Inst* 1987; 78:869–874. 3471995

[51] Li Q Nishijo M Nakagawa H Relationship between urinary Cd and mortality in habitants of a Cd-polluted area: a 22-year follow-up study in Japan. *Chin Med J* 2011; 124:3504–3509. 22340168

[52] Zeng X Jin T Jiang X Effects on the prostate of environmental Cd exposure—a cross-sectional population study in China. *Biometals* 2004; 17:559–565. 15688864 10.1023/b:biom.0000045739.89653.67

[53] Gray MA Centeno JA Slaney DP Environmental exposure to trace elements and prostate cancer in three New Zealand ethnic groups. *Int J Environ Res Public Health* 2005; 2:374–384. 16819092 10.3390/ijerph2005030001

[54] Yaman M Atici D Bakirdere S Comparison of trace metal concentrations in malign and benign human prostate. *J Med Chem* 2005; 48:630–634. 15658876 10.1021/jm0494568

[55] Sarafanov AG Todorov TI Centeno JA Prostate cancer outcome and tissue levels of metal ions. *Prostate* 2011; 71:1231–1238. 21271612 10.1002/pros.21339

[56] Sahmoun AE Case LD Jackson SA Cadmium and prostate cancer: a critical epidemiologic analysis. *Cancer Invest* 2005; 23:256–263. 15945511 10.1081/cnv-200055968

[57] Van Wijngaarden E Singer EA Palapattu GS Prostate-specific antigen levels in relation to Cd exposure and zinc intake: results from the 2002 National Health and Nutrition Examination Survey. *Prostate* 2008; 68:122–128. 18044729 10.1002/pros.20668

[58] Huff J Lunn RM Waalkes MP Cd-induced cancers in animals and in humans. *Int J Occup Environ Health* 2007; 13:202–212. 17718178 10.1179/oeh.2007.13.2.202 PMC3399253

[59] Hartwig A Schwerdtle T Interactions by carcinogenic metal compounds with DNA repair processes: toxicological implications. *Toxicol Lett* 2002; 127:47–54. 12052640 10.1016/s0378-4274(01)00482-9

[60] Strumylaite L Kregzdyte R Bogusevicius A Association between cadmium and breast cancer risk according to estrogen receptor and human epidermal growth factor receptor 2: epidemiological evidence. *Breast Cancer Res Treat* 2014; 145:225–232. 24692081 10.1007/s10549-014-2918-6

[61] Itoh H Iwasaki M Sawada N Dietary Cd intake and breast cancer risk in Japanese women: a case-control study. *Int J Hyg Environment Health* 2014; 217:70–77. 10.1016/j.ijheh.2013.03.010 23608001

[62] Nagata C Nagao Y Nakamura K Cd exposure and the risk of breast cancer in Japanese women. *Breast Cancer Res Treat* 2013; 138:235–239. 23358902 10.1007/s10549-013-2414-4

[63] Luckett BG Su LJ Rood JC Cadmium exposure and pancreatic cancer in south Louisiana. *J Environ Public Health* 2012; 2012:180186. 23319964 10.1155/2012/180186 PMC3540786

[64] Kriegel AM Soliman AS Zhang Q Serum cadmium levels in pancreatic cancer patients from the East Nile Delta region of Egypt. *Environ Health Perspect* 2006; 114:113–119. 16393667 10.1289/ehp.8035 PMC1332665

[65] Park RM Stayner LT Petersen MR Cadmium and lung cancer mortality accounting for simultaneous arsenic exposure. *Occup Environ Med* 2012; 69:303–309. 22271639 10.1136/oemed-2011-100149 PMC4633087

[66] Demir N Turksoy VA Kayaalti Z The evaluation of arsenic and Cd levels in biological samples of cases with lung cancer. *Tuberk Toraks* 2014; 62:191–198. 25492816

[67] Verougstraete V Lison D Hotz P Cadmium, lung and prostate cancer: a systematic review of recent epidemiological data. *J Toxicol Environ Health B Crit Rev* 2003; 6:227–255. 12746140 10.1080/10937400306465

[68] Brys M Nawrocka AD Miekos E Zinc and Cd analysis in human prostate neoplasms. *Biol Trace Elem Res* 1997; 59:145–152. 9522056 10.1007/BF02783239

[69] Lindegaard PM Hansen SO Christensen JE The distribution of cadmium within the human prostate. *Biol Trace Elem Res* 1990; 25:97–104. 1699585 10.1007/BF02990270

[70] Waalkes MP Cadmium carcinogenesis. *Mutat Res* 2003; 533:107–120. 14643415 10.1016/j.mrfmmm.2003.07.011

[71] Rani A Kumar A Lal A Cellular mechanisms of cadmium-induced toxicity: a review. *Int J Environ Health Res* 2014; 24:378–399. 24117228 10.1080/09603123.2013.835032

[72] Lutzen A Liberti SE Rasmussen LJ Cadmium inhibits human DNA mismatch repair in vivo. *Biochem Biophys Res Commun* 2004; 321:21–25. 15358209 10.1016/j.bbrc.2004.06.102

[73] Pierron F Baillon L Sow M Effect of low-dose cadmium exposure on DNA methylation in the endangered European eel. *Environ Sci Technol* 2014; 48:797–803. 24328039 10.1021/es4048347

[74] Asara Y Marchal JA Carrasco E Cadmium modifies the cell cycle and apoptotic profiles of human breast cancer cells treated with 5-fluorouracil. *Int J Mol Sci* 2013; 14:16600–16616. 23941782 10.3390/ijms140816600 PMC3759927

[75] Hartwig A Mechanisms in Cd-induced carcinogenicity: recent insights. *Biometals* 2010; 23:951–960. 20390439 10.1007/s10534-010-9330-4

[76] Johnson MD Kenney N Stoica A Cadmium mimics the in vivo effects of estrogen in the uterus and mammary gland. *Nat Med* 2003; 9:1081–1084. 12858169 10.1038/nm902

[77] Stoica A Katzenellenbogen BS Martin MB Activation of estrogen receptor-alpha by the heavy metal cadmium. *Mol Endocrinol* 2000; 14:545–553. 10770491 10.1210/mend.14.4.0441

[78] Akesson A Julin B Wolk A Long-term dietary cadmium intake and postmenopausal endometrial cancer incidence: a population-based prospective cohort study. *Cancer Res* 2008; 68:6435–6441. 18676869 10.1158/0008-5472.CAN-08-0329

[79] Peng L Huang Y Zhang J Cadmium exposure and the risk of breast cancer in Chaoshan population of southeast China. *Environ Sci Pollut Res Int* 2015; 22:19870–19878. 26289334 10.1007/s11356-015-5212-1

